# Remarkable Physical and Thermal Properties of Hydrothermal Carbonized Nanoscale Cellulose Observed from Citric Acid Catalysis and Acetone Rinsing

**DOI:** 10.3390/nano10061049

**Published:** 2020-05-29

**Authors:** RH Fitri Faradilla, Lucian Lucia, Marko Hakovirta

**Affiliations:** 1 Department of Food Science and Technology, Faculty of Agriculture, Universitas Halu Oleo, Kota Kendari, Sulawesi Tenggara 93232, Indonesia; rh.fitri@gmail.com; 2 Department of Forest Biomaterials, North Carolina State University, Raleigh, NC 27695, USA; lalucia@ncsu.edu

**Keywords:** nanofibrillated cellulose, hydrochar, catalyst, hydrothermal carbonization

## Abstract

Citric acid (CA) was used for the hydrothermal carbonization (HTC) of cellulose nanofiber and found to exert remarkable effects on the chemistry and physical aspects of the product distribution. More specifically, the morphology, yield, elemental and proximate composition, chemical functional groups, thermal properties and surface properties of the resultant hydrochars were studied extensively. The morphological properties of the final char were the singularly most surprising and unique finding of this study. The cellulose nanofiber hydrochars were contrasted to hydrochars from bleached softwood pulp, having a similar composition with the former, to pinpoint the role of nano-dimensions. Without the presence of CA, the pulp hydrochar lacked several of the spherical dimensions shown in the nanocellulose; however, and unexpectedly, the presence of CA caused a homogenization of the final product distribution for both samples. Finally, thermally stable and high surface area hydrochars were obtained when the hydrochar was rinsed with acetone.

## 1. Introduction

Solid hydrochar obtained from hydrothermal carbonization (HTC) has been articulated as a promising biomass carbon sequestration platform that can be utilized for a wide range of applications including catalysis, absorbency, soil rejuvenation and bioenergy [1,2,3,4]. Hydrochars mimic the burning characteristics of sub-bituminous coal and can potentially substitute about 10–25% of coal needed for generating electricity [5,6]. Moreover, the activated hydrochars have excellent CO_2_ capture properties that can play an important role in reducing carbon emission from fossil fuel combustion [7]. In addition, carbon spheres produced from HTC have an outstanding capacity as anode material in lithium ion batteries [8].

Hydrochars are also considered environmentally friendly materials because they are made from renewable sources with relatively low energy inputs and generated about 24% higher net-energy than biochars from slow pyrolysis [5]. The carbonization can be done without drying biomass which usually has high moisture content, thus lowering drying and energy costs [9]. Compared to alternative carbonization protocols, e.g., pyrolysis, HTC produces carbon-rich materials at low temperatures (180–250 °C) and short times (1–12 h) [10]. The exothermic nature of the HTC reaction serendipitously reduces extramural energy demands to maintain temperature [11]. In addition, virtually no toxic gases are emitted because any are dissolved in the liquid media [9].

The final properties of hydrochars are affected by the raw materials, temperatures, reaction times, pressures, and catalyst/additives [12]. Cellulose has been one of the most studied raw materials in HTC with respect to the effect of the latter mentioned experimental variables on the properties of its hydrochars [13]. However, unlike pyrolysis, little is known when nanocellulose, a biomaterial of pronounced importance, undergoes HT carbonization. In a preliminary study from this group, the effect of nanocellulose fibers on the structure and porosity of its hydrochar was studied which demonstrated an optimum time and temperature for HTC [14]; however, it was concluded that more work was necessary to understand the role of nano-dimensions on the properties of hydrochars, especially with respect to the possibility of employing a catalyst to expedite the overall scope and efficiency of HT reactions. 

The addition of acid is known to have a catalytic effect on hydrochar formation. Acid has been shown to act as a catalyst for dehydrating carbohydrates into 5-hydroxymethyl furfural (HMF), an intermediate product of solid char [15]. Other studies found that the properties of hydrochars are highly affected by the acidity of the feedstocks [16,17,18]. Acids such as citric acid, which is safe, inexpensive, and commonly used in HTC, modify the density, shape, particle size, color, surface area, and chemical functional groups of hydrochars [16,17,18].

In the current research, citric acid was used to catalyze the HTC of cellulose nanofibers. The effects of the acid treatment on the morphology, yield, elemental and proximate composition, chemical functional groups, combustion and thermal properties, and surface properties of the resultant hydrochars were studied and compared with hydrochars from bleached softwood pulp, which had a similar composition with the former. This was done to identify the role of the nano-dimension during HTC. Interestingly, substantial changes were obtained when the hydrochar was rinsed with acetone. The discovery of acetone rinsing as a means to obtain thermally stable and high surface area hydrochars was quite unexpected. 

## 2. Methods 

Never dried cellulose nanofiber (CNF) was generously provided by Stora Enso Corporation, Helsinki Finland. Commercial bleached softwood pulp was manufactured in the Southeast US. Citric acid, acetone, and methanol were purchased and used as obtained from Fisher Scientific. 

Hydrothermal carbonization (HTC) was performed at 250 °C for 3 hours in a PARR reactor (PARR Model 4843) without stirring. Two different types of feedstock were used, *viz.*, CNF and softwood pulp (pulp). HTC was done under two different conditions: without the addition of citric acid or in water only (W) and with the addition of citric acid (CA). Anhydrous citric acid was added at an acid and solid feedstock ratio of 1:1. The resultant hydrochar was separated from the liquid by vacuum filtration. Water was used to rinse the hydrochar to completely removing remaining water-soluble materials. The hydrochar was air-dried after which a part of the air-dried hydrochar was rinsed with acetone and methanol to remove tar. These hydrocars are referred to acetone-rinsed hydrochars. 

## 3. Analysis

Morphology of the hydrochars was characterized using the FEI Verios 460L field-emission scanning electron microscope (SEM). At least 10 images per sample were taken at the magnification of 1000 x to determine sphere size distribution. The diameter of the spheres was measured using Image-J. The yield of hydrochars was calculated by the ratio of the dry weight of water rinsed hydrochar to the dry weight of the feedstock. The concentration of C, H, and N of hydrochar and tar was analyzed using the Perkin Elmer Corporation’s model 2400, series II elemental analyzer (Waltham, MA, USA) in a pure oxygen environment. The oxygen concentration was calculated by difference. The higher heating value (HHV) was determined using Dulong’s equation [19]. Total carbon (TC) and total organic carbon (TOC) of the final liquid were analyzed using the TOC Analyzer (Shimadzu PC-Controlled Total Organic Carbon Analyzer, Japan). The pH of the initial materials and final liquid were determined using a pH meter. FTIR spectra of the hydrochars were taken over 4000–600 cm^−1^ using the Perkin Elmer Frontier FTIR spectrometer with a Universal ATR sampling accessory. Thermogravimetric analysis (TGA) (TA-TGA Q500) was used to determine proximate composition, combustion, and thermal properties of the hydrochars. TGA was run under air and nitrogen gas flow at a temperature of 30–800 °C using a temperature ramp of 10 °C min^−1^. The concentration of volatile materials was calculated by subtracting 100% to the weight loss (%) during the dehydration stage and the remaining weight (%) at the end of the TGA under nitrogen gas flow. Ash content was the remaining weight (%) at the end of the TGA under airflow. Fixed carbon was calculated by difference (100 − (moisture + volatile materials + ash)). Characteristic temperature (dehydration stage, devolatilization and combustion stage, char combustion stage, ignition temperature (T_i_), maximum combustion temperature (T_m_), and burn out temperature (T_b_) of hydrochars were analyzed following a known method [20]. Brunauer, Emmett and Teller (BET) surface area and porosity of hydrochars were measured by Micromeritics Gemini VII-Surface Area and Porosity. Hydrochars were first degassed using Micromeritics SmartPrep-Programmable Degas System with nitrogen gas at 200 °C for 3 hours. The analysis was done with nitrogen as the adsorptive at saturation pressure of 771.319 mmHg and −195.85 °C. 

## 4. Results and Discussion

**Figure 1:**
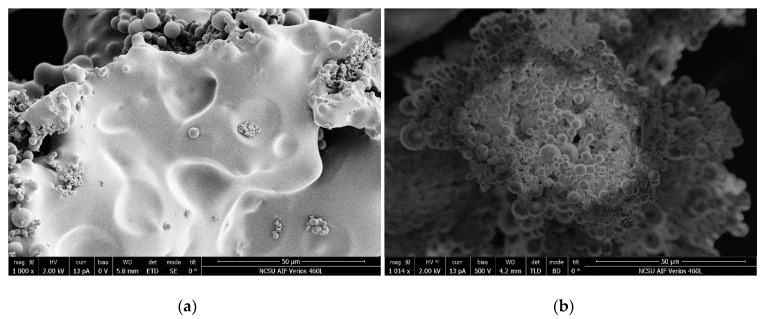
SEM images of cellulose nanofiber in water (CNF-W) hydrochar before and after rinsing with acetone, (**a**) Without acetone rinsing; (**b**) With acetone rinsing.

Hydrothermal carbonization of cellulose nanofiber (CNF) and softwood pulp (pulp) in two different environments was undertaken in the water only and the citric acid solution regimes. The resultant hydrochars were thoroughly washed with water to remove any remaining liquid attached to the solid hydrochars. Figure 1a shows the SEM image of the CNF-W-hydrochar after water rinsing and air drying. There are two distinctive features in the image: spheres and a continuous matrix. The continuous matrix has a smooth surface while a portion of the spheres are covered by the matrix. This continuous matrix was suspected to be tar which was readily removed by organic solvents. It was rinsed with acetone using the Soxhlet apparatus for maximum tar removal. The acetone rinsing treatment had a significant effect on the morphology of the hydrochars as shown in Figure 1b. The smooth and continuous matrix was completely gone, and a greater number of spheres were clearly visible. 

**Figure 2:**
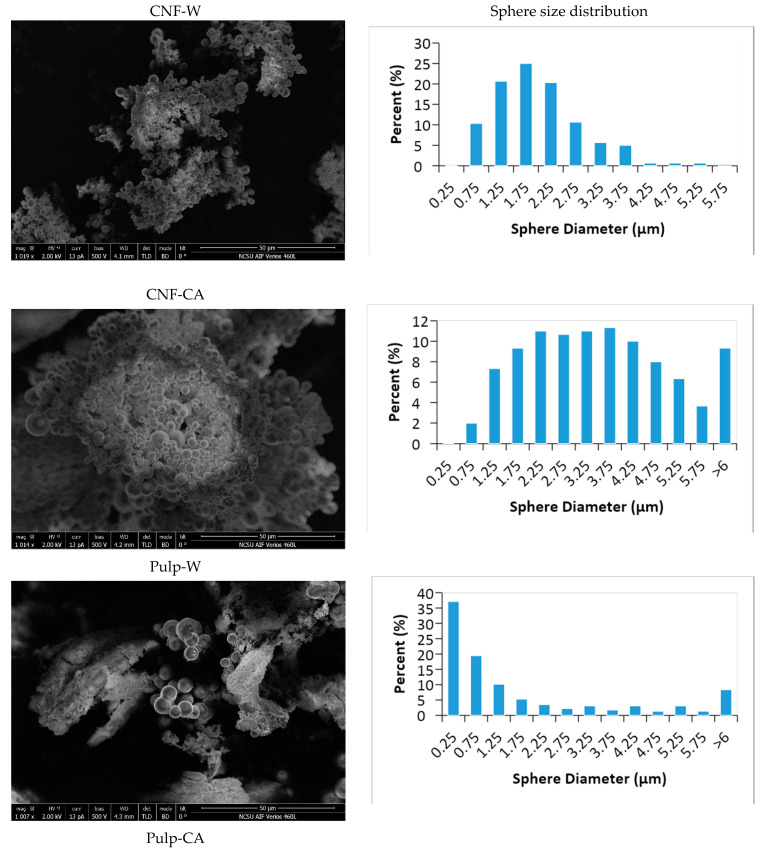

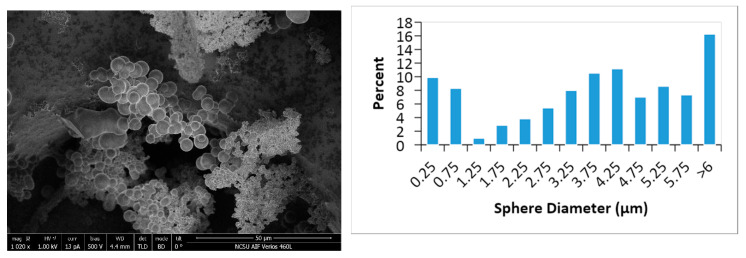
SEM images of acetone rinsed hydrochars and the sphere size distribution.

All hydrochar samples were subsequently rinsed with acetone to reveal the true appearance of the chars (Figure 2). Remarkably, a greater number of spheres were observed in CNF-W compared to pulp-W. Pulp-W still had a bulky log-like structure, while the CNF-W mostly composed of spheres and continuous coral-like structure. Higher magnification of SEM images of the hydrochars is available as Supplementary Materials. A microsphere-like structure is the typical form of hydrochars obtained from monosaccharides [21]. It can be safely concluded that the nano-dimension of CNF provides a sufficiently high enough surface area to expedite the hydrolysis of cellulose into simple sugars which therefore leads to a greater number of carbon spheres produced in CNF-W than in the pulp-W. 

The addition of citric acid increased the diameter of carbon spheres in both the cellulose nanofiber and softwood pulp samples. In addition, the carbon with a bulky and log-like structure that was observed in Pulp-W was no longer evident in pulp -CA. The appearance of pulp-CA hydrochar was almost like the cellulose nanofiber hydrochars (CNF-W and CNF-CA). The presence of citric acid in the mixture likely induced the hydrolysis of cellulose into soluble oligomers and glucose, which underwent dehydration, condensation, and polymerization reactions [22,23] to form carbon spheres. It was also reported that the diameter of microspheres became larger when HTC was run in the presence of HCl [22].

### 4.1. Yield and Compositions

Despite its effect on size and homogeneity of spheres, citric acid did not greatly affect the yield of hydrochar from cellulose nanofiber (Table 1). However, the yield of hydrochar from softwood pulp was greater; 32.2% for pulp-W and 50.5% for pulp-CA. The effect of acid on the yield of hydrochars was found to not be uniform in past reports. For example, a number of accounts reported an adverse effect of acid on yield, albeit the type of acids—strong acids, such as HCl and H_2_SO_4_ [22,24] or weak acids such as citric acid and acetic acid [25,26]. However, it was found that the concentration of H_2_SO_4_ positively correlated to the yield of hydrochars from rice husk [27]. They argued that the acid favors dehydration and carbonization of the carbon precursors [27]. In contrast, acid (H_2_SO_4_) inhibits the conversion of 5-hydroxymethylfurfural (HMF) into solid carbon [24]. It has been claimed that acid facilitates the formation of levulinic acid, which is marked by a lower pH and higher levulinic content in the final liquid [24]. This pH reduction was not observed in this study. The pH of the final liquid of the water-treated sample and CA-treated sample was similar, despite differences in the initial pH (Table 1). Therefore, citric acid might induce hydrolysis, as evidenced by the larger sphere size (Figure 2), and also catalyze dehydration and carbonization.

Elemental composition was not affected by treatment variables, types of feedstock, presence of an acid, and acetone rinsing. Hydrochars and tars had a carbon content in the range of 65–70%, nitrogen = 0.06–0.10%, and oxygen = 25–33%. Relative to the hydrochars, the tar was mostly composed of carbon, which made it insoluble in water. The similarity of the elemental composition of hydrochars, regardless of the type of feedstock, has also been previously reported [28]. It was found that the carbon content of hydrochars from lignin, cellulose, D-xylose, and wood meal was in the range of 63–75% [28]. The absence of the effect of citric acid on the carbon ratio is also in line with a past report [29].

The total carbon (TC) and total organic carbon (TOC) of the liquid gathered at the end of HTC were measured (Table 1). Most of the carbon in the liquid was organic, which may consist of sugars and their derivatives, organic acids, furanoids, and phenolics [30]. Interestingly, although liquid pH was similar, the amount of TC and TOC of CA-treated samples was higher than in the water-treated samples. Carbon from citric acid may contribute to an increase in TC and TOC because according to similar work [31], HTC of citric acid alone does not produce a solid carbon. 

**Figure 3:**
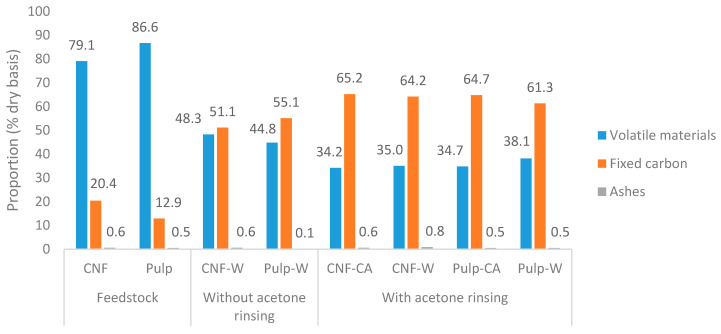
Proximate composition of feedstocks and hydrochars.

**Figure 4:**
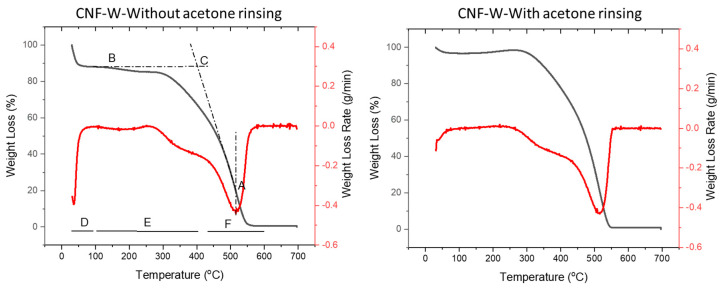
Thermogravimetry-digital thermogravimetry (TG-DTG) curves of cellulose nanofiber (CNF) hydrochars.

In the proximate compositional analysis, the fraction of volatile materials, fixed carbon, and ashes of feedstocks and hydrochars were determined. In Figure 3, the feedstocks were mainly composed of volatile materials and only ~ 13–20% of fixed carbon. As expected during HTC, cellulose undergoes carbonization that significantly reduced volatiles and increased the fraction of fixed carbon. Washing the hydrochars with acetone further increased the proportion of fixed carbon that demonstrates that tarry substances were more volatile than the hydrochars, although the two had a comparable elemental composition (Table 1). The thermogravimetry-digital thermogravimetry (TG-DTG) curves in Figure 4 also confirm this phenomenon.

The effects of types of feedstock and acid treatment were not significant on the proximate content of the hydrochars. Acetone rinsed hydrochars possessed 34–38% volatile materials, 61–65% fixed carbon, and 0.5–0.8% ash. The level of fixed carbon of hydrochars was relatively high since it is larger than the fixed carbon content of lignite, which is approximately 15–50% on a dry basis [32,33]. The proportion of ash in hydrochars was similar to the feedstocks, which indicates that most of the ash was not soluble in the HTC liquid. 

### 4.2. ATR-FTIR Analysis

**Figure 5:**
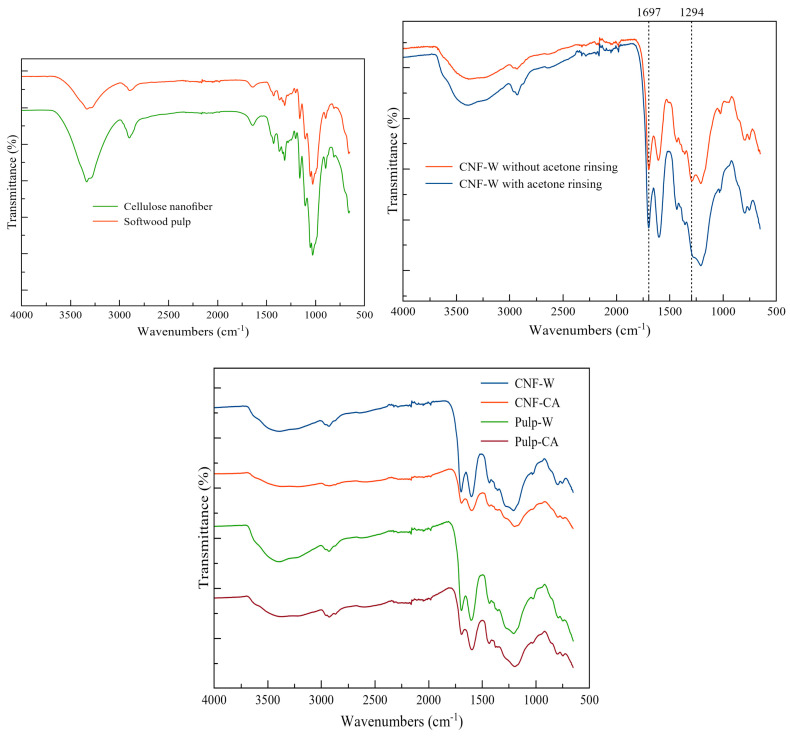
ATR-FTIR spectra of feedstocks and hydrochars.

Attenuated total reflectance Fourier transform infrared (ATR-FTIR) spectra of the feedstocks and hydrochars are presented in Figure 5. The spectra of the cellulose nanofiber and softwood are alike, which point to the similarity in the chemical compounds of the two materials. Rinsing the hydrochar with acetone reduced the carbonyl groups at 1697 cm^−1^ and aromatic ester C–O stretching at 1294 cm^−1^. The reduction on these peaks has also been previously reported [34] when the hydrochars were washed with tetrahydrofuran. These functional groups could be the product of an intermediate reaction of cellulose carbonization. It has been previously stated that rinsing swine hydrochar with acetone removes –OCH_3_ groups and soluble intermediates, such as residual monomers and degradation products of hemicellulose and cellulose [35]. After acetone rinsing, hydrochars from all samples had similar ATR-FTIR spectra, which confirm the nano-dimensions of CNF and the fact that the acid treatment did not modify the chemical structure of the hydrochars. The hydrochars had peaks corresponding to the carboxylic groups at 1600 cm^−1^ and 1434 cm^−1^, carbonyl groups at 1697 cm^−1^, C–O bonds at 1300–1000 cm^−1^, and hydroxyl groups at 3700–3000 cm^−1^ [36]. The signal at 1600 cm^−1^ indicates the presence of aromatic C=C with a benzene skeleton [37].

### 4.3. Combustion and Thermal Properties

TG-DTG curves of hydrochars from CNF are presented in Figure 4. Other hydrochars have a similar pattern of TG-DTG curves with that shown in Figure 4. The major difference between the TG-DTG curves of unrinsed and acetone rinsed hydrochars was that the unrinsed hydrochar has a weight loss at 100–260 °C, while mass reduction other than moisture in the rinsed hydrochar started at ~ 250 °C. This shows that acetone rinsing removed the volatile tarry substances. The proportion of these volatile tarry substances was in the range of 2–3%. 

A summary of the dehydration, devolatilization, and combustion temperatures of feedstocks and hydrochars is presented in Table 2. The dehydration stage (area D in Figure 4) of the feedstocks occurred at a higher temperature than the hydrochars, which indicates a weak interaction between hydrochar and water. CNF and pulp as feedstocks are rich in hydrophilic cellulose, while the hydrochars are carbon species with low H/C and O/C ratios that make them more hydrophobic. The devolatilization and combustion stage (area E in Figure 4) occurred in two steps in unrinsed hydrochars and only one step in rinsed hydrochars. As explained earlier, the tarry substances, which were removed by acetone rinsing, were volatile and degraded at a lower temperature (started at 95–105 °C) than the char. Acetone rinsing is recommended especially when the hydrochars are subjected to high temperature (higher than 100 °C) because the unrinsed hydrochars will release fume.

Table 2 also lists the characteristic temperatures of the samples. The effect of acid treatment on the char characteristic temperatures was more pronounced than the effect of cellulose nano-dimension (Table 2). Citric acid increased T_i_ (point C in Figure 4), T_m_ (point A in Figure 4), and T_b_ of the hydrochars. High T_i_ is favorable for fuel to reduce the risk of fire and explosion [38]. A harsher condition seems to produce hydrochars with higher ignition temperature and longer combustion process [20,38].

### 4.4. Surface Properties

**Figure 6:**
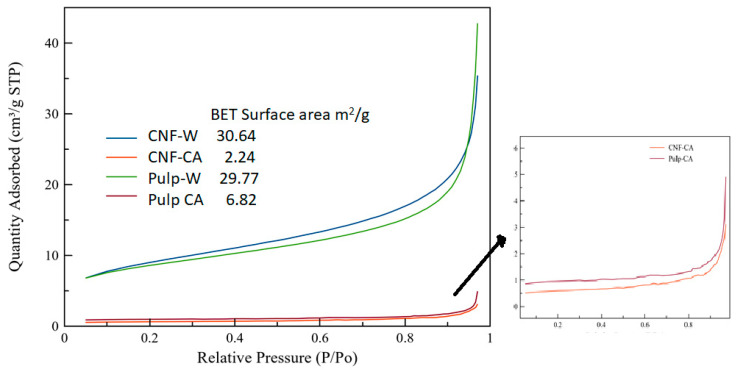
N_2_ adsorption isotherms and BET (Brunauer, Emmett and Teller) surface area of hydrochars.

The N_2_ adsorption isotherms and BET surface area of hydrochars are presented in Figure 6. Based on the classification of physisorption isotherms of IUPAC Technical Report [39], the adsorption isotherm curves of the hydrochars followed Type II isotherms. Type II isotherms usually occur in nonporous or microporous adsorbents [39]. The average pore size of the hydrochars was also in the range of 50–90 nm, which indicated the presence of macropores. 

The BET surface area of CNF-W and pulp-W was approximately 30 m^2^ g^−1^ which is higher than the BET surface area of similar hydrochars without acetone rinsing (9 m^2^ g^−1^). The acetone rinsing seemed to play a major role in increasing the surface area since the rinsing removed the tar that covers the surface of hydrochars [40]. It has also been reported that the BET surface area increased due to the organic solvent rinsing of hydrochars [34]. The results also indicate that there was no appreciable difference between BET surface area of hydrochar from cellulose nanofiber and softwood pulp. The BET surface area and quantity of adsorbed nitrogen were more affected by acid treatment. As can be seen in Figure 6, acid treatment reduced the BET surface area and the quantity of adsorbed nitrogen dramatically. This agrees with previous work where an increase in acid concentration reduces the surface area [41]. It was argued that the acid triggers the growth of the carbon spheres; thus, more large spheres were present in hydrochars than tiny spheres. This argument is also in line with our SEM results in Figure 2, where the size of carbon spheres in CNF-CA and pulp-CA were larger than in the CNF-W and pulp-W. 

## 5. Conclusions

The present work provides a simple account of the important role of citric acid to catalyze the hydrothermal carbonization of cellulosics with respect to nanodimensions. It was found that bulk cellulose provided tubular arrays of hydrochar byproducts which were very distinct to the nanoscopic spheres generated from nanocellulose. However, the inclusion of citric acid appeared to level the playing field by homogenizing both bulk and nano-cellulose morphological product distributions. In addition, acetone rinsing of nanocellulosic hydrochars appeared to increase the surface area because it removed the surface tar. Unrinsed hydrochar had a weight loss at over a range of 160 °C, while mass reduction other than moisture in the rinsed hydrochar started at ~250 °C that demonstrated that acetone rinsing removed the volatile tarry substances (2–3%).

## Figures and Tables

**Table 1:** Yield, elemental composition, and pH.

Sample Codes	Yield (%)	Hydrochar											Tar			Liquid			Initial pH
		Before Acetone Rinsing				After Acetone Rinsing													
		C (%)	H (%)	N (%)	O (%)	C (%)	H (%)	N (%)	O (%)	H/C	O/C	HHV (MJ kg^−1^)	C (%)	H (%)	N (%)	TC (mg L^−1^)	TOC (mg L^−1^)	pH	
CNF-W	42.8	62.90	3.88	0.10	33.12	66.42	4.09	0.10	29.39	0.06	0.44	23.0	70.14	5.54	0.08	5558	5537	2.94	3.59
CNF-CA	44.5					65.10	4.29	0.06	30.55	0.07	0.47	22.7				15,010	14,929	2.95	1.87
Pulp-W	32.2	70.73	4.39	0.13	24.75	66.95	4.20	0.06	28.79	0.06	0.43	23.5	70.08	5.76	0.09	7938	7917	2.78	6.34
Pulp-CA	50.5					68.11	4.29	0.10	27.50	0.06	0.40	24.2				22,820	22,352	2.70	1.68

HHV: higher heating value; TC: total carbon; TOC: total organic carbon.

**Table 2:** Thermogravimetric analysis of feedstocks and hydrochars.

Samples	Dehydration Stage (°C)	Devolatilization and Combustion Stage (°C)	Char Combustion Stage (°C)	Characteristic Temperature (°C)		
				Ti	Tm	Tb
Feedstocks						
CNF	178	220–300	520–580	518.5	558.8	573.0
		330–450				
Pulp	125	290–450	490–570	515.9	543.7	555.6
Without acetone rinsing						
CNF-W	75	95–265	440–580	401.1	515.9	558.2
		265–440				
Pulp-W	93	105–260	430–620	403.7	510.8	583.9
		260–430				
With acetone rinsing						
CNF-W	78	240–430	430–560	415.0	513.4	553.7
CNF-CA	108	260–470	470–630	440.4	540.0	624.8
Pulp-W	92	240–430	430–590	404.2	509.6	577.6
Pulp-CA	85	260–443	443–593	450.8	532.5	587.8

Ti: ignition temperature; Tm: maximum combustion temperature; Tb: burn out temperature; CNF: cellulose nanofiber; Pulp: softwood pulp.

## Supplementary Materials

Supplementary materials are available online at https://www.mdpi.com/2079-4991/10/6/1049/s1.
