# Supplementary material for: Remarkable Physical and Thermal Properties of Hydrothermal Carbonized Nanoscale Cellulose Observed from Citric Acid Catalysis and Acetone Rinsing
Source: Nanomaterials (Basel). 2020 May 29;10(6):1049. doi: 10.3390/nano10061049 (PMC7352704; doi:10.3390/nano10061049)
Supplement: Supplementary file 1 [file nanomaterials-10-01049-s001.pdf]

CNF-W

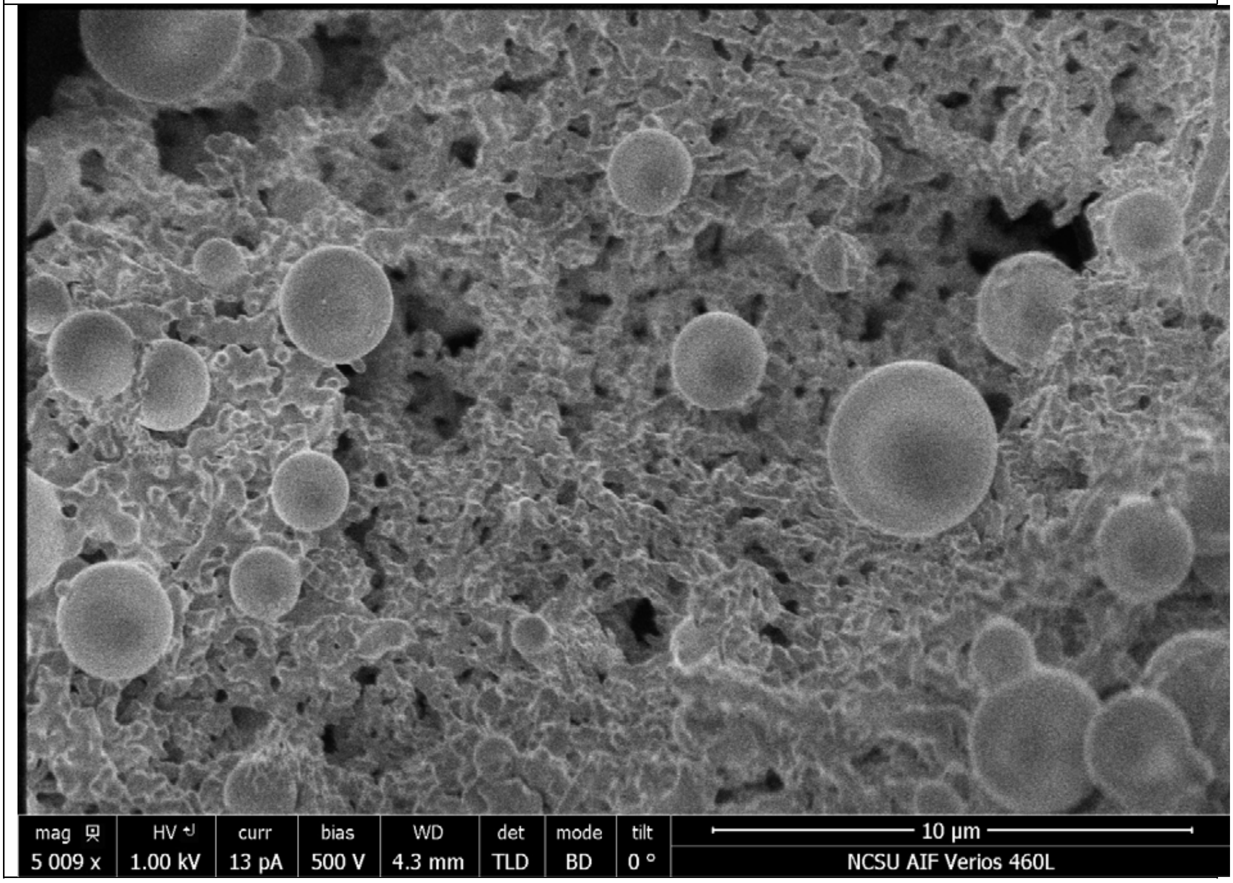

mag
5 009 x
HV
1.00 kV
curr
13 pA
bias
500 V
WD
4.3 mm
det
TLD
mode
BD
tilt
0 °
10 µm
NCSU AIF Verios 460L

CNF-CA

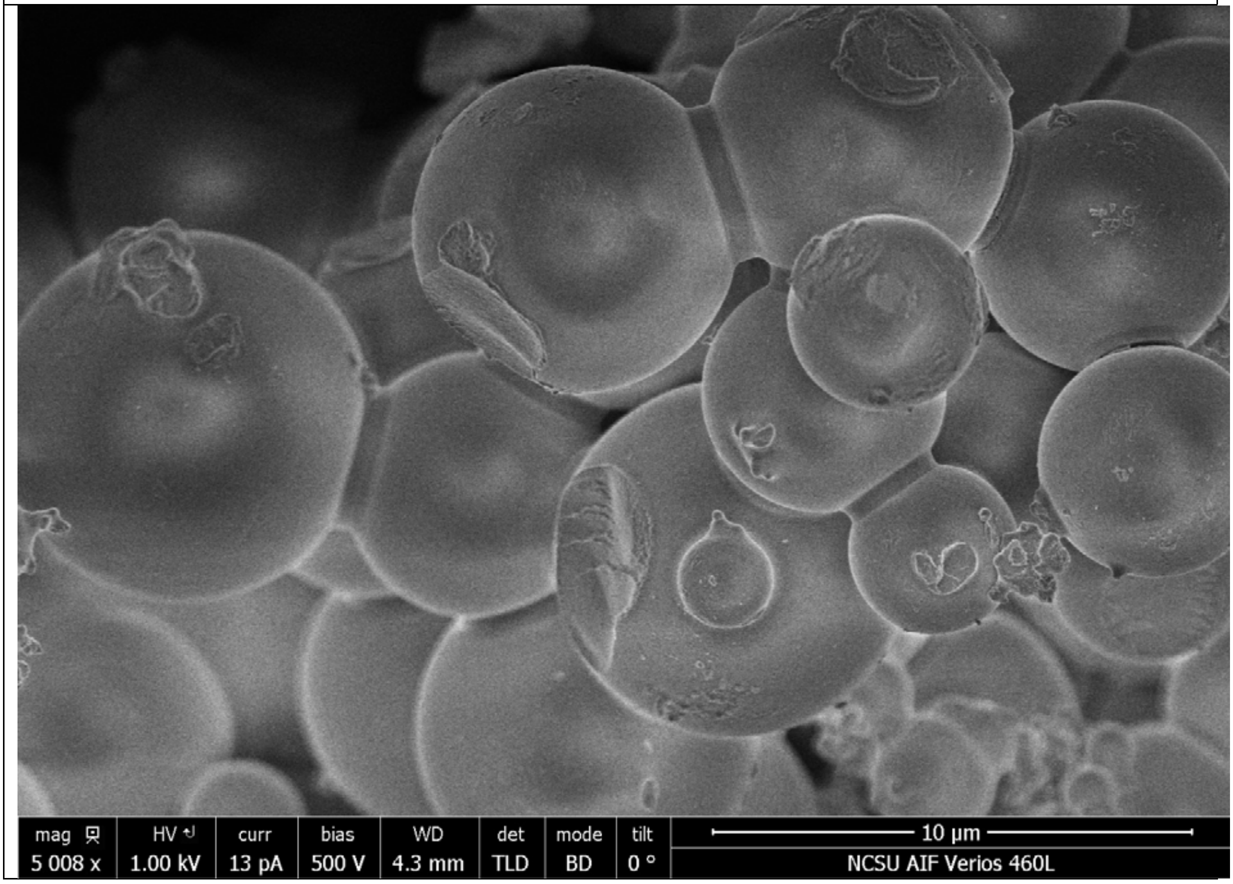
mag
5 008 x
HV
1.00 kV
curr
13 pA
bias
500 V
WD
4.3 mm
det
TLD
mode
BD
tilt
0 °
10 µm
NCSU AIF Verios 460L

Pulp-W

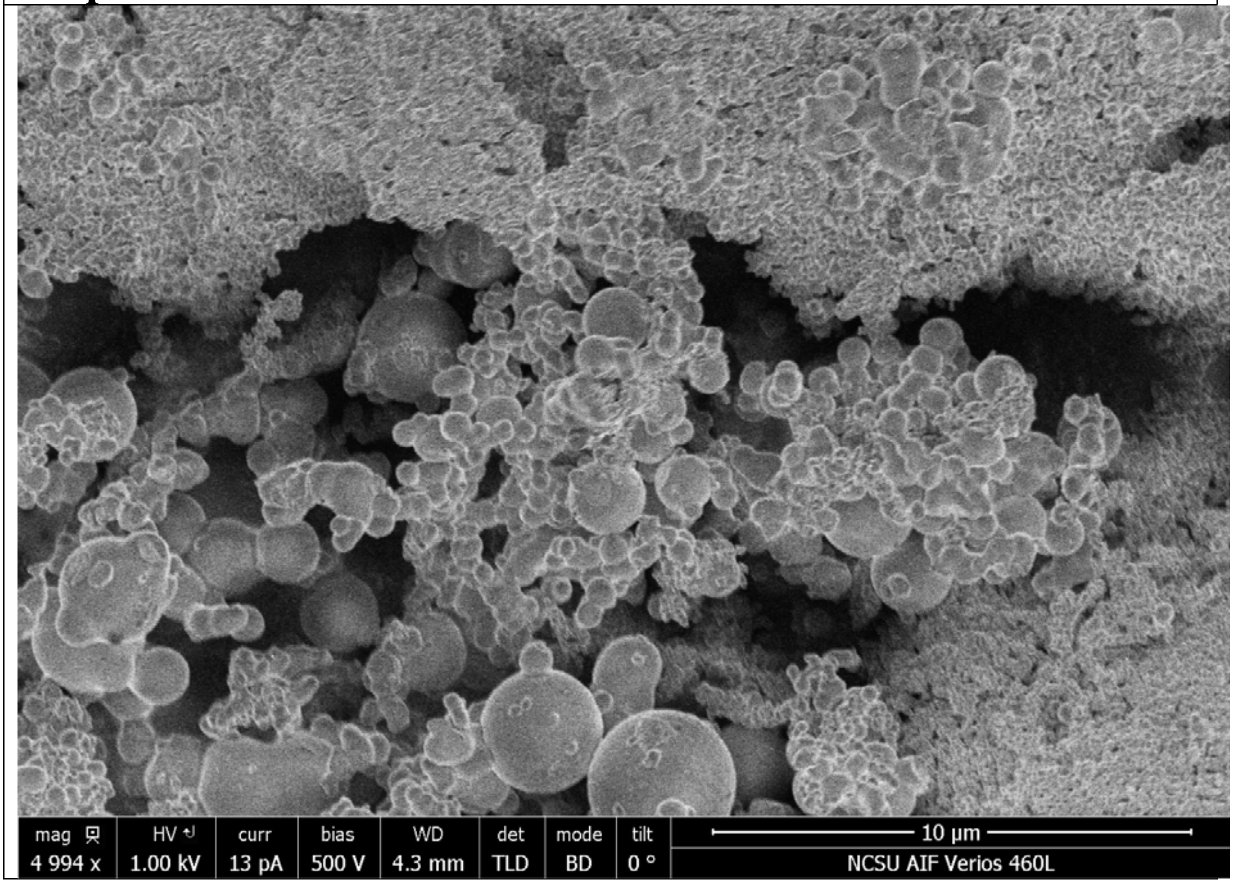
mag
4 994 x
HV
1.00 kV
curr
13 pA
bias
500 V
WD
4.3 mm
det
TLD
mode
BD
tilt
0 °
10 µm
NCSU AIF Verios 460L

Pulp-CA

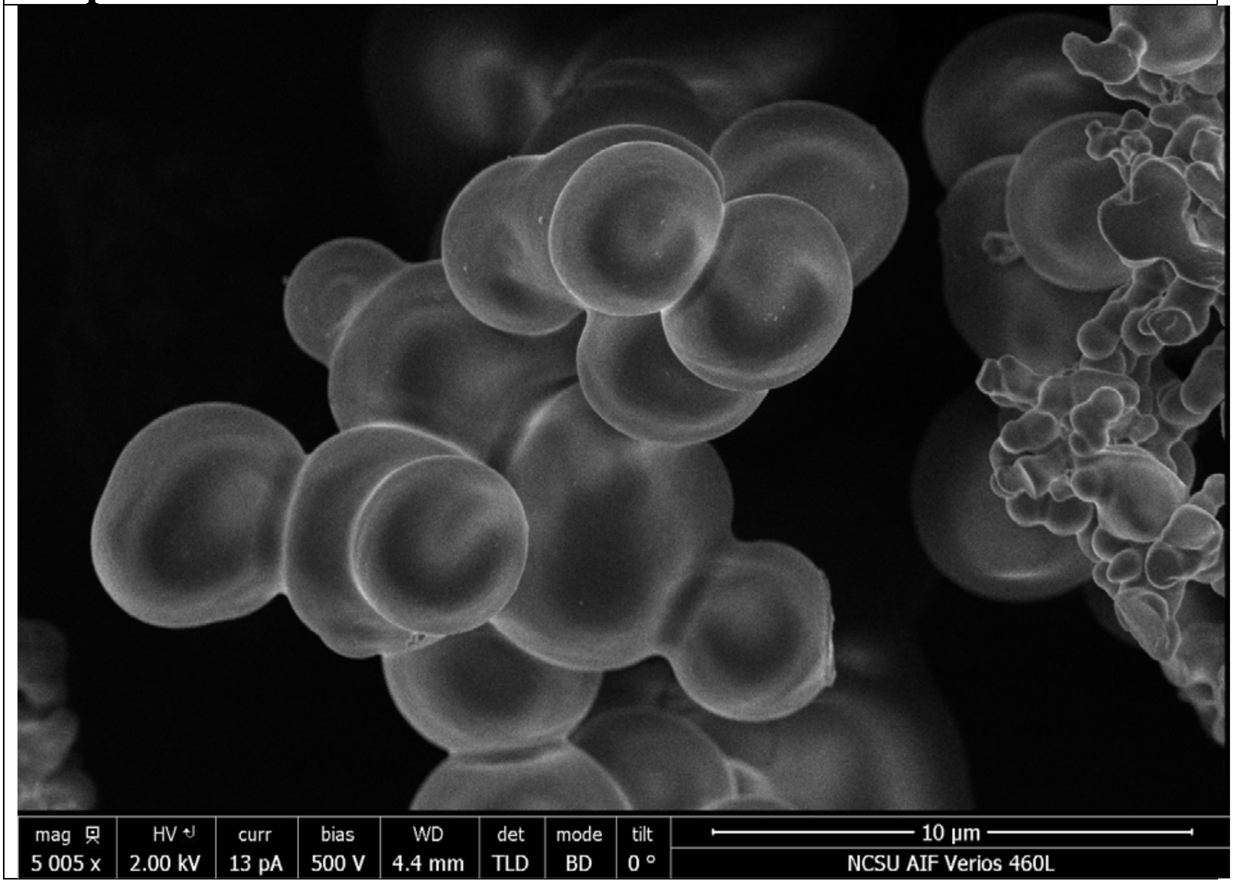
mag
5 005 x
HV
2.00 kV
curr
13 pA
bias
500 V
WD
4.4 mm
det
TLD
mode
BD
tilt
0 °
10 µm
NCSU AIF Verios 460L
